# Link Investigation of IEEE 802.15.4 Wireless Sensor Networks in Forests

**DOI:** 10.3390/s16070987

**Published:** 2016-06-27

**Authors:** Xingjian Ding, Guodong Sun, Gaoxiang Yang, Xinna Shang

**Affiliations:** 1 Information School of Beijing Forestry University, Beijing 100083, China; bjfudxj@163.com (X.D.); recessburton@gmail.com (G.Y.); shangxinna@buu.edu.cn (X.S.); 2 College of Information Technology, Beijing Union University, Beijing 100101, China

**Keywords:** wireless sensor networks, low-power wireless link, link performance, forest monitoring

## Abstract

Wireless sensor networks are expected to automatically monitor the ecological evolution and wildlife habits in forests. Low-power links (transceivers) are often adopted in wireless sensor network applications, in order to save the precious sensor energy and then achieve long-term, unattended monitoring. Recent research has presented some performance characteristics of such low-power wireless links under laboratory or outdoor scenarios with less obstacles, and they have found that low-power wireless links are unreliable and prone to be affected by the target environment. However, there is still less understanding about how well the low-power wireless link performs in real-world forests and to what extent the complex in-forest surrounding environments affect the link performances. In this paper, we empirically evaluate the low-power links of wireless sensors in three typical different forest environments. Our experiment investigates the performance of the link layer compatible with the IEEE 802.15.4 standard and analyzes the variation patterns of the packet reception ratio (PRR), the received signal strength indicator (RSSI) and the link quality indicator (LQI) under diverse experimental settings. Some observations of this study are inconsistent with or even contradict prior results that are achieved in open fields or relatively clean environments and thus, provide new insights both into effectively evaluating the low-power wireless links and into efficiently deploying wireless sensor network systems in forest environments.

## 1. Introduction

Recent years have seen a rapid growth of using wireless sensor networks to monitor the physical world [1,2,3,4,5,6]. A wireless sensor network consists of many tiny sensors deployed in the field; these sensors can detect temporal-spatial physical signals and transmit their measurements to end users through wireless (radio) links. The wireless links are capable of interconnecting the sensors into a communication network and then allow end users both to remotely observe the sites that interest them and to obtain a great deal of sensory data that contribute to more accurate decision making. As a promising instrument for collecting data about the physical world, therefore, wireless sensor networks have been adopted in a variety of monitoring applications, including the natural environment, the military field, urban traffic, building structure health, and so on.

Wireless communication greatly facilitates the monitoring system deployment and potentially saves human resources in data collection, yet it has to be carefully considered in reality [7,8,9,10,11]. In practical wireless sensor network systems, the energy efficiency is a key performance metric, because it dominates the longevity by which the system can serve end users; the radio chip or the transceiver equipped in wireless sensors is the most energy hungry component. To date, in both industry and academia, researchers and engineers en masse have moved to the low-power wireless communication, aimed to fundamentally reduce the energy consumption of sensors in communications. The IEEE802.15.4 [12], for instance, is a typical low-power wireless standard, which usually works at the 2.4-GHz or the 868/915-MHz ISM band, and supports a 250-kbps data rate with the energy budget of only at most a few milliwatts. To be compatible with the IEEE 802.15.4 standard, more and more radio chips, such as TR1000, CC1000, CC2420, etc., are fostered and widely adopted in practice. From the past engineering experiences, the involvement of low-power wireless links is an inevitable choice in wireless sensor networks aimed at long-term monitoring.

Such low-power radio links, however, often lead to unreliable communication or data transportation in wireless sensor networks; and sometimes, they are rendered with high dynamics, especially when they are deployed in harsh environments with more obstructions or interference sources. Inherently, the radio signal propagation will be reflected, scattered and diffracted by the surrounding objects [13,14]; these effects are more significant and unpredictable for low-power wireless links [15,16]. In particular, the signals emitted by the low-power, low-cost transceivers of wireless sensors are easily distorted by the overspreading internal and ambient noise. Though wireless sensor networks and traditional wireless networks share several link characteristics, low-power links are more lossy and time-varying in coverage [17]. Therefore, understanding the low-power radio link well is very critical and indispensable to design effective and efficient wireless sensor networking protocols, such as medium-accessing control (MAC), routing and network topology control. This paper attempts to investigate the performance of the low-power wireless link in forest environments. Our study is motivated mainly by two facts.

First, large-scale and long-term forest monitoring has emerged in recent years as an effective way by which domain scientists can comprehensively investigate the forestry resources, as well as the in-forest environmental parameters and then accurately model the natural evolution related to forests [6,18,19,20]. For instance, combining the forest data and the weather data, environment scientists can study the relationship between climate change and the growth of trees or they can evaluate the function of trees in conserving soil and water. Wireless sensor networks, however, are facing new challenges in forest monitoring due to the complex forest environment. Different from the open and obstacle-free scenarios, the forest site involves densely- and irregularly-distributed trees, shrubs and vegetation, all of which will not only significantly degrade the performance of wireless links, but also make it more difficult to model the link dynamics.

Second, although researchers have realized the unique challenge of low-power wireless links in complex environments and several works have been proposed to evaluate the performance of low-power radio signals, they either consider only the physical-layer characteristics or study the link behaviors just from one or a few aspects. The physical-layer results are helpful to understand the path loss ratio of the wireless link and to optimize the design of the transceiver and modulation schemes, but they hardly provide adequate insights into improving the whole low-power wireless network because at runtime, the program cannot obtain the precise in situ path loss information to carry out the real-time adjustment of protocol parameters. Some prior works analyze the link characteristics in terms of link reliability, the signal strength or other link metrics. Nevertheless, the observations and analyses are mostly achieved in open-field scenarios or indoor environments with less obstacles. The performance of low-power links in forests and their effects on upper-layer protocols remain uncertain or unknown to some extent.

The remainder of paper is organized as follows. Section 2 describes the experimental sites and configurations. Section 3 presents the evaluation results of a single low-power link, and Section 4 evaluates the links of a small-scale wireless sensor network. Section 5 summarizes the experimental observations of this paper with insightful remarks. Section 6 introduces major works related to ours. Finally, Section 7 concludes this paper.

## 2. Experimental Methodologies

This section will depict the forest sites in which we conduct experiments, the sensor platform, as well as the wireless standard and the evaluation metrics. We will introduce the detailed procedures and configurations of the experiment in later sections.

### 2.1. Study Sites

**Figure 1:**
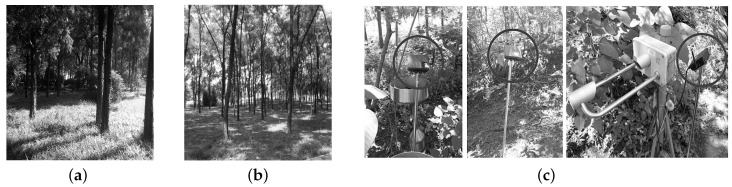
Three typical forest environments. (**a**) Campus forest; (**b**) Bajia Rural Park forest; (**c**) Jiufeng Mountain forest.

In this paper, we consider three city forest environments to evaluate the performance of low-power radio links. The first forest is the Bajia Rural Park forest of Beijing; the second one is a forest inside our university campus; the third one is on a mountain of the Jiufeng National Forest Park of Beijing. We will later call the three forests the Bajia forest, the Campus forest and the Jiufeng forest, respectively. The three forest sites are different in terms of the tree density, the vegetation distribution and the understory terrain. Figure 1a,b shows the Campus and the Bajia forests, and their tree densities are about 17/100 m^2^ and 20/100 m^2^, respectively. The understory of the Campus forest, involving sparse short shrubs, is covered by lush vegetation, which is 40 cm in height on average, while the understory of the Bajia forest is covered by a thin vegetation layer with an average height of 23.6 cm.

The Jiufeng forest site is shown in Figure 1c. In comparison with the two above in-city forests, the Jiufeng forest, along a hillside, has a very complex and diverse understory, including shrubs, weeds, withered branches and foliage and even bare soil; the tree density is about 25/100 m^2^. In fact, the Jiufeng forest site is a typical forest terrain of concern by hydrologists and forestry scientists, and that is the reason that we choose the Jiufeng forest as a study site. Additionally, Figure 1c also shows the placement of wireless sensor nodes marked with circles, and the experiments in the Bajia and the Campus forests use the same wireless sensor nodes, as well. Each sensor node is installed on the top of a steep bracket, around 1.2 m away from the ground.

In experiments under all three sites, the temperature was 20 °C on average, and the wind stayed moderate, swinging the forest gently. In particular, neither people nor other moving ground objects passed through the study sites. We did not find any significantly detectable IEEE 802.11 (WiFi) and IEEE 802.15.1 (Bluetooth) signals of 2.4 GHz in the surroundings. We use a Bosch’s handheld laser ranging device of DLE40 to determine the distance between the sending and the receiving nodes and the size of the vegetation.

### 2.2. Experimental Setup

#### 2.2.1. Wireless Standard and Sensor Node

We adopt Crossbow’s TelosB sensor nodes in our experiments. The TelosB sensor node, formerly called the TmoteSky node [21], is an integrated platform including a TI MSP430 microcontroller, a ChipCon CC2420 radio transceiver [22] of 2.4 GHz and an onboard antenna. Compatible with the IEEE 802.15.4 standard, the TelosB node is very popular in practice, because it provides end users with a set of external AD/DAand GPIOports that can easily interface with other sensing and actuating devices. The TelosB node transmits data with a power of at most 1 mW; so the link formed by two TelosB nodes is a typical low-power link. The TelosB node can be powered either by two AA batteries or by its built-in USB interface connecting to a host computer. One disadvantage of TelosB node is its low-gain antenna printed on the board, which diminishes the radio communication range. In our experiment, therefore, we expand the TelosB node with an undirected external antenna of 3 dBi, as shown in Figure 1c, besides encapsulating the TelosB node with the aluminum package.

#### 2.2.2. Software Configuration

The TelosB nodes in the experiments run the NesC codes. NesC is a programming language specially devised for wireless embedded devices, and it is commonly used together with the TinyOS, a light-weight and open-source operating system developed by UC Berkeley. To evaluate the link performance, we let nodes exchange messages according to a pre-configured plan. In detail, the program on each node controls the message sending with a specific period of time and records necessary information once a message is received; the message includes the message sequence, the RSSI (received signal strength indicator) and the LQI (link-quality indicator) measurements, as well as the receiving time. The RSSI and the LQI are two simple link quality metrics and can both be directly returned by the CC2420 radio chip, without needing any application-level computation.

The experimental data are delivered to the laptop, which connects with the TelosB node via a USB port and runs a serial-port listening program of Java, thereby being able to receive all of the link information sent by the connected TelosB node. Once the data (a record) arrives, the laptop will immediately push it into a local database of MySQL. We will give more details, in later sections, about how to carry out the experiments.

### 2.3. Metrics

We use three basic metrics for evaluating the performance of the low-power links in forests: RSSI, LQI and packet reception ratio (PRR). These three metrics are often used in empirical studies and networking protocol designs.

- PRR: If node *A* sends *n* packets to node *B* and *B* correctly receives m(m≤n) packets, then the PRR of the link from *A* to *B* is equal to $\frac{m}{n}$. Calculated at the receiver side, PRR is often used as a benchmark metric for link reliability in wireless protocol design and operation, especially in routing protocols.
- RSSI: This is a reading calculated by a receiver’s radio chip, which generally is the average of the signal strength of eight-symbol periods. For the TelosB node, which integrates the CC2420 radio chip, the returned RSSI value ranges from −100 dBm to 0 dBm. The RSSI involves not only the received signal, but the background noise, and generally, the received signal is hard to discern from noise when the returned RSSI is lower than −90 dBm.
- LQI: The receiver can measure the strength quality of a successfully received packet by calculating the average correlation of the first eight symbols of this packet. LQI is often used to approximate the chip error rate. The TelosB node produces an LQI value of at most 110 and at least 50.

In this paper, we first conduct experiments under the two in-city forest environments with only a single low-power wireless link formed by two nodes; second, we deploy a small-scale wireless sensor network of ten nodes in a mountain forest environment and investigate the performance of the wireless in-network links.

## 3. Performance of Single Link

### 3.1. Experiment Designs

In the single link experiment under the two forests shown in Figure 1, we denote the two nodes by *A* and *B*, and the two directional links between them are denoted by *A*-*B* (*A* sends data to *B*) and *B*-*A* (*B* sends data to *A*), respectively. In experiments, *A* first broadcasts 600 probing messages to *B* with a period of 10 ms. After the completion of *A*’s probe sending, *B* starts broadcasting 600 probes back to *A*, still with the time interval of 10 ms. When a probe is received by *A* or *B*, the receiver will measure the RSSI and the LQI of this probe by invoking the functions supported by the CC2420 radio chip. To record the link information, each node is connected to a laptop via the USB port. The PRR is calculated at the laptop by the ratio of the successfully received probes and the transmitted probes. Most wireless sensor networks in practice pursue long-term monitoring, so the sensor nodes often use as low transmit powers in communication as possible.

The CC2420 transceiver equipped in the TelosB node supports eight transmit power levels that can be programmed at runtime; they are 0, −1, −3, −5, −7, −10, −15 and −25 in units of dBm, and they are represented in programs with parameters (tx) 31, 27, 23, 19, 15, 11, 7 and 3, respectively. The two last transmit power levels are too weak to form a valid link; we therefore choose the first six levels in experiments. In the experiments, nodes *A* and *B* are placed 4×d meters apart, where d∈{1,2,⋯,12}. The longest distance of the sender and receiver is 48 m in the Bajia experiment, beyond which the links almost always fail in the direction from *A* to *B*, or the direction from *B* to *A*, or both. Note here that in the Campus experiment, the longest distance between the two nodes is set to be 36 m, because it is the longest line-of-sight distance we can obtain there; the laser ranging device in use works only under the line-of-sight scenario. We repeatedly conduct the single-link experiment under different link distances and different transmit power levels.

### 3.2. Effect of Distance and In-Forest Surroundings

**Figure 2:**
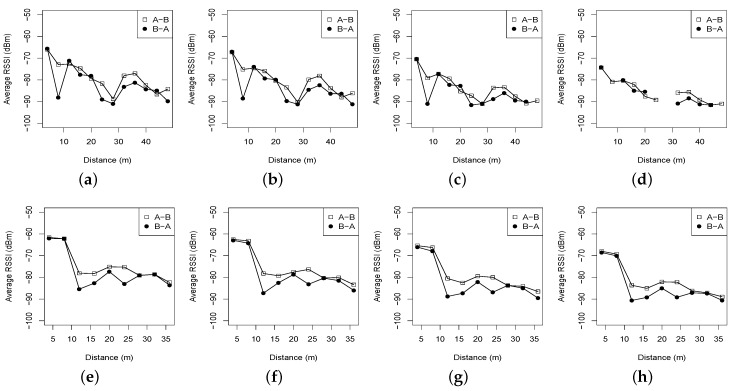
RSSI distributions vs. distances with different transmit powers under two different forests. (**a**) Bajia, tx = 31; (**b**) Bajia, tx = 27; (**c**) Bajia, tx = 19; (**d**) Bajia, tx = 15; (**e**) Campus, tx = 31; (**f**) Campus, tx = 27; (**g**) Campus, tx = 19; (**h**) Campus, tx = 15.

**Figure 3:**
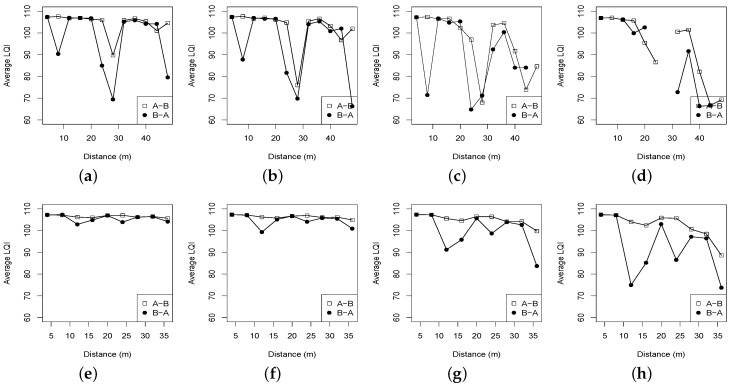
Link-quality indicator (LQI) distributions vs. distances with different transmit powers under two different forests. (**a**) Bajia, tx = 31; (**b**) Bajia, tx = 27; (**c**) Bajia, tx = 19; (**d**) Bajia, tx = 15; (**e**) Campus, tx = 31; (**f**) Campus, tx = 27; (**g**) Campus, tx = 19; (**h**) Campus, tx = 15.

Figure 2 and Figure 3 show the distributions of RSSI measured by the receiving node (from two directions) with four transmit power levels (tx), 31, 27, 19 and 15. We can see that for the two forest environments, the RSSI values of both directions roughly decline as the link distance increases. When the link distance is beyond 48 m in the Bajia forest, or 36 m in the Campus forest, the obtained RSSI is reduced almost to −90 dBm, the sensitivity threshold of the CC2420 transceiver. In addition, with each of the four transmit powers, the Campus and the Bajia forests both experience obvious decreased RSSI when the link distance is not too long; for instance, when tx = 31 and the link distance increases from 4 m to 20 m, the RSSI decreases from −66 dBm to −80 dBm in the Bajia forest and from −62 dBm to −78 dBm in the Campus forest, respectively. However, as the link distance is longer than 20 m with tx = 31 or 16 m with tx = 27, the RSSI of the Campus forest descends gently until no radio carrier can be detected, different from the case in the Bajia forest. Clearly, in forest environments, the RSSI value is not linearly correlated with the communication distance.

**Figure 4:**
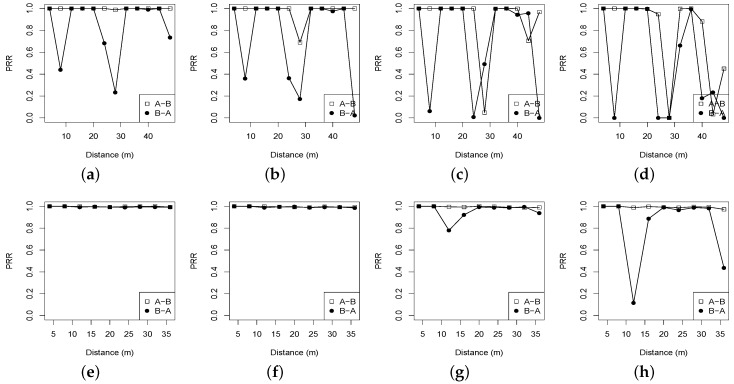
Packet reception ratio (PRR) distributions vs. distances with different transmit powers under two different forests. (**a**) Bajia, tx = 31; (**b**) Bajia, tx = 27; (**c**) Bajia, tx = 19; (**d**) Bajia, tx = 15; (**e**) Campus, tx = 31; (**f**) Campus, tx = 27; (**g**) Campus, tx = 19; (**h**) Campus, tx = 15.

Compared with the RSSI of the Campus forest, the RSSI of the Bajia forest fluctuates more significantly in the descending trend for each transmit power. This observation implies that the reflection and scattering caused by relatively dense vegetation and sparse shrubs (in the Campus forest) do not impact the low-power radio link as much as we had thought; the vegetation or the short shrubs will possibly improve the radio propagation to some degree. Moreover, such an observation is also partially proven in Figure 4, illustrating the PRR dynamics under two forest environments. Note here that there are a few missing points in Figure 2 and Figure 3 because no probes are successively received in the Bajia experiment where the link distance and the transmit power are set to be 28 m and 15, respectively; however, this does not imply that this experimental configuration in forests always cannot produce valid links. Nevertheless, we believe that such a point loss represents the real status of low-power wireless links in forests.

The LQI’s distributions are shown in Figure 3. For the CC2420 transceiver, an LQI value close to 110 indicates a highest-quality packet, and in contrast, the link of around 50 LQI measurement is typically the lowest-quality link [22]. It can be seen from Figure 3 that for each forest environment, the LQI shows a downtrend as the transmit power is reduced from 31 to 15. The LQI of the Bajia forest is pretty much unstable with sharper transitions than that of the Campus forest. For example, with higher transmit powers, 31 and 27, the LQI of the Bajia forest ranges from 70 to 108, while the LQI in the Campus forest from 100 to 108. This observation is almost inconsistentwith the aforementioned RSSI distributions in Figure 2: a small quantity of vegetation in the Campus forest does not necessarily impact the low-power radio link too much. Compared to the RSSI distributions, the LQI shows a relatively moderate descending tendency when the link distance is not too long. In the Bajia forest with tx = 31, for instance, the RSSI shows an exact declining tendency as the link distance is not beyond 24 m, while the corresponding LQI does not change significantly. However, when the transmit power is set to be lower (e.g., 19 and 15) and the link distance increases up to 36 m, the RSSI in the Bajia forest declines gently, while the corresponding LQI decreases sharply. The comparison results here demonstrate different variation patterns of RSSI and LQI.

Though the RSSI and the LQI can roughly profile the link quality in some scenarios, an effective and commonly-used approach of evaluating link quality in practice is to calculate the runtime PRR of a radio link, which is the reception ratio of a set of probing messages that are successively sent or broadcast within a pre-configured period of time. Figure 4 plots the PRR measurements in the two forest environments. In the Campus forest environment with more vegetation and sparse bushes, the link PRR generally performs better, in both directions of the link, than the link PRR of the Bajia forest does. The interesting behavior of the PRR of the Bajia forest is its irregular variations, although in the experiments, two nodes are always line-of-sight, and the link is formed under the canopy without penetrating any bushes or leaves. For example, when tx = 31 in the Bajia forest, the link of a distance of 8 m produces a PRR of 42%, while the link of a distance of 32 m produces a PRR close to 100%. Such an irregular variation also occurs in the Campus forest experiment in which the link is often non-line-of-sight or the receiver is surrounded by laighshrubs, but they are infrequent, even in the scenarios with lower transmit powers, say tx = 19 or tx = 15. Comparing Figure 3 to Figure 4, we can see that the PRR has a variation pattern similar to the LQI, indicating that in forest environments, the link quality in terms of PRR can be probably predicted with the LQI. This will be further investigated in a later section.

**Figure 5:**
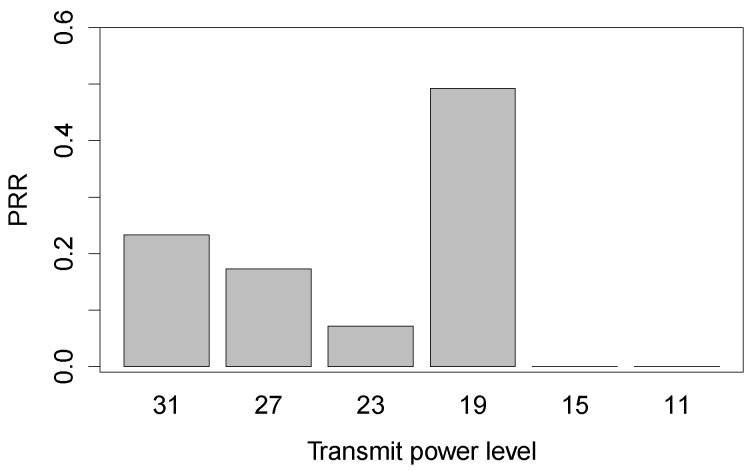
PRR distributions vs. transmit powers of the link B-Aof the distance of 28 m under the Bajia forest.

In the experiments, we find that for a given communication distance, higher transmit powers cannot always lead to higher link quality, not conforming to what has been traditionally deemed in theoretic studies. Figure 5 shows the PRR distributions of link *B*-*A* against transmit powers under the Bajia forest with the link distance of 28 m. As the transmit power decreases from 31 to 23, the corresponding PRR sharply drops from 0.23 to 0.07; however, the transmit power of 19 achieves a relatively higher PRR of 0.49. In our experiments, we fix the sending node and move the receiving node to different positions, yet keeping the same distance, in order to form distance-identical links with different receiver’s directions. Such extra controlled experiments prove that the result of Figure 5 is not rare for in-forest low-power wireless links. The reason behind this might be that higher transmit powers result in a more complex multi-path effect that degrades the radio propagation in the forest environment.

### 3.3. Asymmetry of the Link

**Figure 6:**
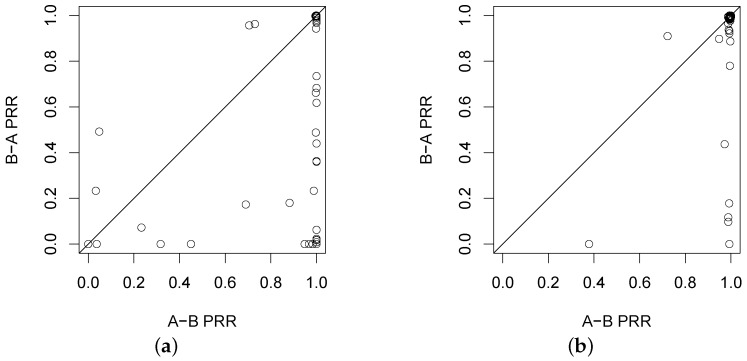
Illustration of link asymmetry under two forest environments. (**a**) Bajia; (**b**) Campus.

The complicated forest environments usually lead to high link asymmetry, that is the qualities of a link on two directions are conspicuously different. Figure 6 shows the difference of the PRRs on two directions of the experimental link, in which all pairs of two-way PRRs are plotted considering different transmit powers and link distances. Clear link asymmetry can be seen in Figure 6. Previous empirical evaluations in the open field show that the link of higher quality or the link of lower quality has better symmetry. However, such a result cannot well hold in our experiment, especially in the Bajia forest: e.g., the PRR of link *B*-*A* varies too much even though the PRR of link *A*-*B* almost keeps at 100%. In the Campus forest, the links with PRR close to 100% are rendered with better symmetry than their counterparts in the Bajia forest. A later section will continue to discuss the time-varying property of link asymmetry.

**Figure 7:**
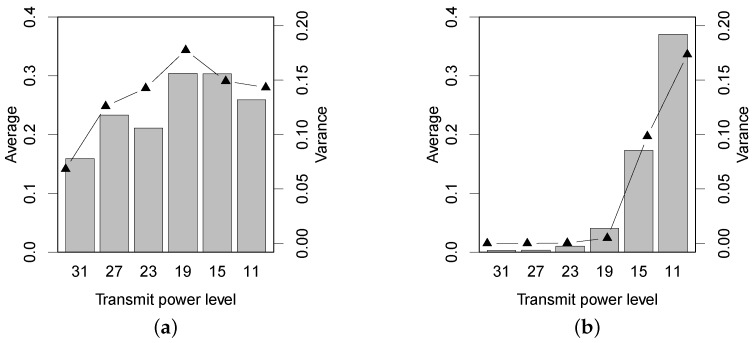
The asymmetry ($D_{prr}$) of PRR under two different forest environments. (**a**) Bajia; (**b**) Campus.

We use the absolute difference of the two-way PRRs of a link, denoted by $D_{prr}$, to quantitatively evaluate the link asymmetry in forests with different experimental settings. For a given transmit power, in detail, we calculate the average and the variance of all of the $D_{prr}$ measurements under different link distances. In total, the average and the variance of the $D_{prr}$ profile are the distribution and the fluctuation of link asymmetry, respectively. Figure 7 shows the PRR asymmetry. In the Bajia forest, the link asymmetry increases as the transmit power level is tuned from 31 down to 19. Noticeably, the average and the variance of $D_{prr}$ both decline when the transmit power ranges from 15 to 11. Prior studies [23,24] find that the low-power wireless link tends to be relatively symmetric when the link quality is very low or very high and is significantly asymmetric when the link quality (the receiver resides in the gray region) is intermediate. However, the result of Figure 7a does not effectively support their findings, because in our experiment, the PRR of long-distance links with lower transmit powers is often close to zero simultaneously in two directions; the $D_{prr}$ values are subsequently weakened.

In the Campus forest, the $D_{prr}$ measurements differ much over transmit power levels. Under the three higher power levels, 31, 27 and 23, the links almost keep symmetric: the PRRs on two directions are both close to 100%, which is also demonstrated in Figure 4. The link asymmetry in the Campus forest starts experiencing a rising trend when the transmit power is lower than 19. By comparing the results of two forests, we find that the in-forest shrubs and short trees (in the Campus forest) will unnecessarily abate the symmetry of low-power wireless links. Additionally, we cannot conclude a significant grey region for the links in the Campus forest experiment.

**Figure 8:**
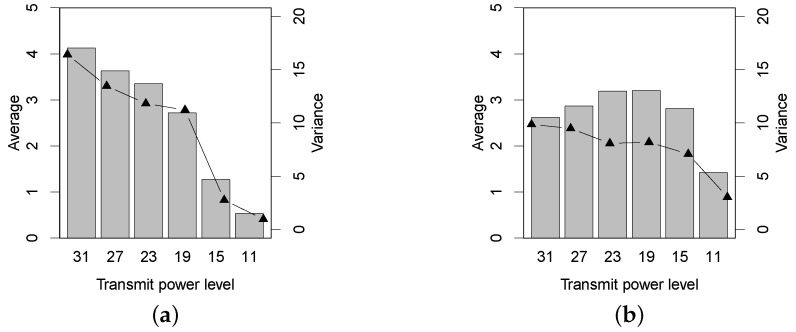
The asymmetry ($D_{rssi}$) of RSSI under two different forest environments. (**a**) Bajia; (**b**) Campus.

**Figure 9:**
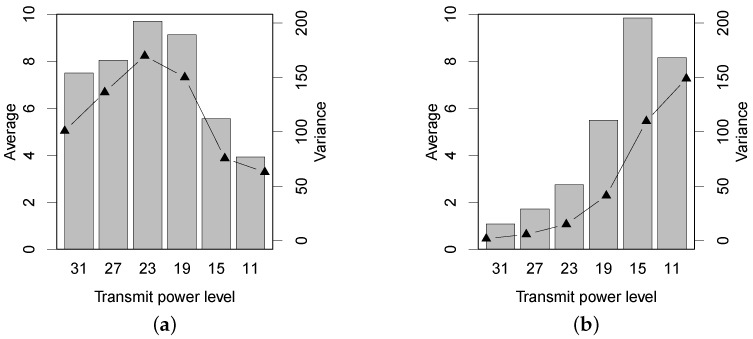
The asymmetry ($D_{lqi}$) of LQI under two different forest environments. (**a**) Bajia; (**b**) Campus.

Besides the PRR, we also use the two-way RSSI and LQI absolute differences of a link, denoted by $D_{rssi}$ and $D_{lqi}$, respectively, to evaluate the link asymmetry, and the according results are plotted in Figure 8 and Figure 9. When the transmit power decreases, in Figure 8, the $D_{rssi}$ in the Bajia forest stably keeps going down, while the $D_{rssi}$ in the Campus forest totally goes down with a little fluctuation. Figure 9 shows the LQI asymmetry for the two forests. It is interesting to note that the average and the variance of $D_{lqi}$ have variations similar to the $D_{prr}$ of Figure 7, especially in the Campus forest environment. To some extent, we can practically resort to the LQI asymmetry, rather than the RSSI asymmetry, to predict the link asymmetry for resource-efficiency purposes.

### 3.4. Propagation Analysis

**Figure 10:**
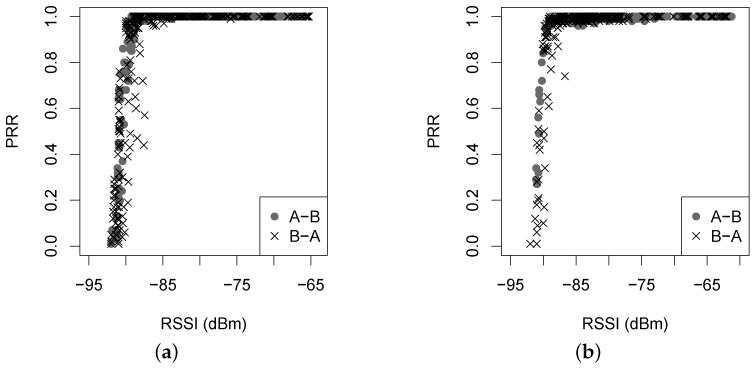
The PRR versus RSSI under two different forest environments. (**a**) Bajia; (**b**) Campus.

**Figure 11:**
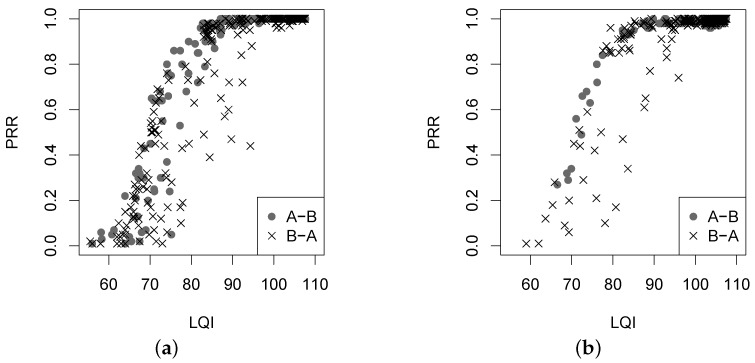
The PRR versus LQI under two different forest environments. (**a**) Bajia; (**b**) Campus.

We first examine the PRR against the RSSI and the LQI. In experiments, two nodes *A* and *B* form the link with two directions, *A*-*B* and *B*-*A*, for a given link distance and a given transmit power. Figure 10 and Figure 11 give the correlation of the PRR and the RSSI and LQI, respectively.

It is clear in Figure 10 that the correlation between PRR and RSSI varies with an obvious transition in the two forest environments. When the RSSI is greater than −87 dBm, the PRR is always beyond 90%, indicating a desirable link; when the RSSI is less than −92 dBm, the PRR is close to zero; however, when the RSSI ranges from −92 dBm to −87 dBm, the PRR is hard to predict. Note here that the sensitivity of the CC2420 radio chip used in experiments is −90 dBm. In the vicinity of such a threshold of sensitivity, the background noise might dominate so often that the PRR not only keeps lower, but also varies irregularly in a large range. In Figure 11, the PRR variation against the LQI also experiences a transition, which is not sharper than that in Figure 10. Comparing the two sub-figures in Figure 11, it can be found that most points in Figure 11b are located on the top right, where the PRR and the LQI are both larger. Recall that in Figure 3, the link in the Campus forest (especially in the direction *A*-*B*) produces higher LQI measurements than that in the Bajia forest. According to the PRR vs. LQI distribution, we have that an LQI larger than 96 can lead to a link with PRR beyond 90%, but when the LQI is less than 96, the PRR varies greatly within 90% and zero. For instance, in the Bajia forest, for the LQI of 75, the corresponding PRR may be equal to 5% or 85%, and for the PRR of 60%, the LQI may be equal to 70 or 90.

Though the link is very dynamic in forest environments, studying the propagation of low-power wireless radios in forests are still helpful to configure relatively reliable parameters in simulation experiments. The radio signal suffers from attenuation during propagation, i.e., the signal strength generally decays, according to Equation (1), as the distance between sender and receiver increases. In Equation (1), *d* is the physical link distance, $P_{0}$ is the received signal power of the receiver one meter away from the sender and *n* is the parameter that weighs the signal attenuation on communication distance. The key job of modeling the propagation attenuation is to determine *n*. In this paper, we consider the RSSI measurement as the received signal strength and transform Equation (1) into Equation (2).

(1) $$P=P_{0}\;d^{-n}$$

(2) $$P\left(\mathrm{dBm}\right)=P_{0}\left(\mathrm{dBm}\right)-10\times n\times \log_{10}\;d$$

**Figure 12:**
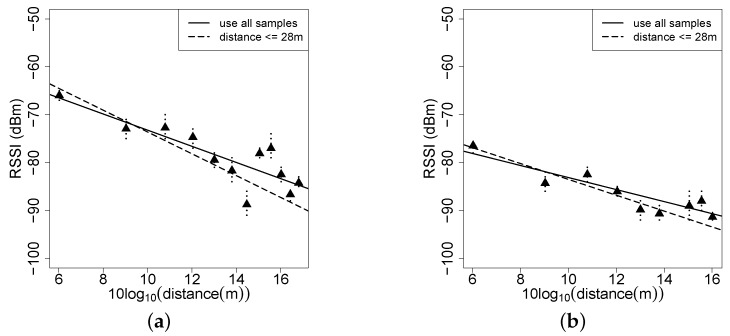
RSSI distance fitting in the Bajia forest environment. (**a**) tx = 31; (**b**) tx = 11.

We treat the “$10\log_{10}\;d$” to be a factor and deduce a linear regression model (i.e., the linear least square) to fit parameter *n*; the results for the Bajia forest are shown in Figure 12. We find that the attenuations are different from transmit power to transmit power. We here give results with the highest and the lowest transmit powers (31 and 11, respectively). In Figure 12, the small dots represent the RSSI distribution and the triangles the mean of RSSI measurements. We give two types of linear fits: the solid line is obtained according to the RSSI in all Bajia experiments and the dashed line according to the RSSI in Bajia experiments with a link distance not greater than 28 m. Such a choice is determined by the fact that if the link distance is set be be longer than 28 m, the RSSI almost reaches to −90 dBm; according to the analysis in Figure 10, the RSSI may be inaccurate to evaluate a link with a distance longer than 28 m.

When the transmit power level is set to 31, from Figure 12a, we obtain $P_{0}=-50.839$ and n=2.277 with the RSSI samples with a link distance no larger than 28 m and $P_{0}=-56.359$ and n=1.687 with all of the RSSI samples. For the transmit power level of 11, in Figure 12b, we get $P_{0}=-66.966$ and n=1.654 with the RSSI samples with a link distance no larger than 28 m and $P_{0}=-70.558$ and n=1.256 with all RSSI samples. From Figure 12, it can been seen that under relatively low transmit power, the RSSI decays slowly as the link distance increases. This numerical result indicates that for in-forest wireless sensor networks, the higher transmit power cannot always overwhelm the lower one for any link distance in terms of link quality; so, we might adopt lower transmit power levels to prolong the network lifetime, while meeting the link quality requirement. Although such fitting results alone cannot achieve a universally sound path loss model, they provide approximated parameters for the simulation-based study of the target wireless sensor network deployed in forests.

## 4. Performance of In-Network Links

### 4.1. Deployment and Experiment Designs

**Figure 13:**
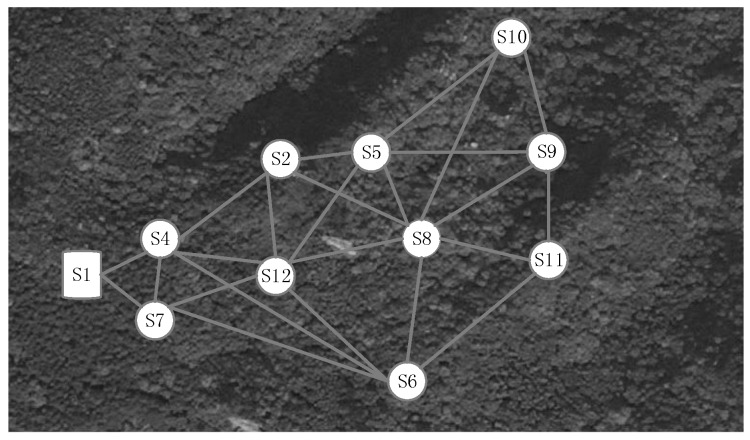
Overview of a ten-node wireless sensor network deployed in the Jiufeng forest (only a part of the wireless links is shown here for clarity).

Besides the evaluation of the dynamics of a single link, we deploy a small-scale wireless sensor network in the Jiufeng National Forest Park, Beijing, China, to achieve a more comprehensive understanding of low-power links in the forest environment. Figure 13 shows the deployment involving ten TelosB sensor nodes, as well as a sink node; and Figure 1c shows the sensor placement and the in-forest terrain characteristics. The ten sensor nodes, labeled with S2∼S12 (excluding S3, due to its damage), are not deployed randomly; they are placed at most 0.2 m away from ten grid-powered hydrology-monitoring devices that have been mounted and maintained by the Water and Soil Conservation Department of our university according to specific standards for ecology monitoring. As Figure 1c shows, such an experimental deployment with designated locations of sensor nodes can better simulate real-world wireless hydrology-monitoring sensor network applications. For all of the pairs of sensor nodes with available links, the maximum link distance is 8.85 m, and the minimum one is 2.2 m. Any pair of nodes is almost non-line-of-sight; between both of which there exists irregular tree leaves, branches, shrubs and even tree trunks. Each sensor node is driven by a pair of AA batteries and sends data with the maximum transmit power (1 mW). The sink node, labeled with S1, is placed at the rim of the sensor network area, and it collects and stores all of the link information returned by the sensor nodes.

In the experiment, each sensor node periodically discovers its neighborhood every two minutes by broadcasting a set of successive probing messages. If a probing message is successfully received, an acknowledgment message will be immediately replied by the receiver to the sender. By doing so, each sensor node can know its neighborhood: who its neighbors are and the link qualities in terms of the PRR, the RSSI and the LQI. In detail, every two minutes, the sender broadcasts ten successive probes with the interval of 50 ms, by which the PRR can be deduced at the receiver side by calculating the ratio between the number of successfully received probes and the total number of probes sent by the sender. Noticeably, we do not impose a globally-synchronized clock for all nodes, because doing so will consume extra network resources and make it complicated to control the neighbor discovering. To collect the link information for the analysis purposes, we use the CTP [7] (Collection Tree Protocol, a de facto routing protocol for wireless sensor networks) to deliver the link information recorded by each sensor node to the sink node. Every two minutes, the sensor node first determines its neighbors and the corresponding link qualities by back-and-forth message exchanges and then pushes the link information of relevance into a single timestamped packet, which will be transported to the sink node over the CTP path. We carried out a two-hour experiment in the middle of August 2015. Note here that if a node cannot determine its forwarders, CTP will first stop the transportation service at this node, drop the corresponding application traffic and then go to re-discover the neighborhood; such a handling process of CTP results in a few packets not being able to be delivered to the sink in our experiment. That is the major reason that several later figures involve a small quantity of missing values. In fact, CTP involves a link estimation component, called the *4-bit link estimator* . However, it provides the link quality estimation from the point of view of routing, i.e., it is designed mainly to facilitate the forwarding decision; therefore, such a link estimator cannot reliably reflect the actual link status.

In the Jiufeng experiment, we investigate all of the link information recorded and find that the total patterns of the PRR, the RSSI, the LQI and the symmetry of these links are similar to their counterparts in the single link experiment, merely with lower measurements. We hence do not describe the performance of each in-network link of the Jiufeng experiment. Instead, we focus on deeply investigating the time-varying characteristics of the in-network low-power wireless links in a real-world forest environment.

### 4.2. PRR of In-Network Links

**Figure 14:**
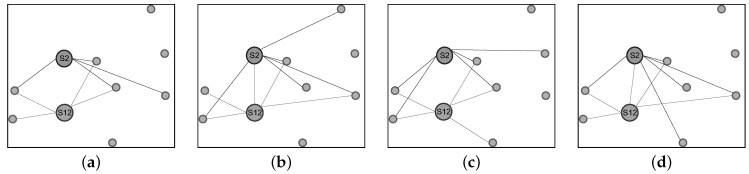
Temporal variation of the topology of the Jiufeng wireless sensor network (shown here are only links incident to nodes S2 and S12). (**a**) The 10th minute; (**b**) The 20th minute; (**c**) The 30th minute; (**d**) The 40th minute.

Figure 14 demonstrates the temporal variation of two nodes’ neighborhoods over four sequential time points. For clarity, in Figure 14, we present only two sensor nodes, S2 and S12, and retain only at most five incident links (neighbors) for each of S2 and S12. Although in our experiment, a sensor node only chooses at most five PRR best neighboring nodes as its neighbors, the variation of the PPR of incident links shown in Figure 14 demonstrates that the topology of the low-power network obviously varies with time increasing.

**Figure 15:**
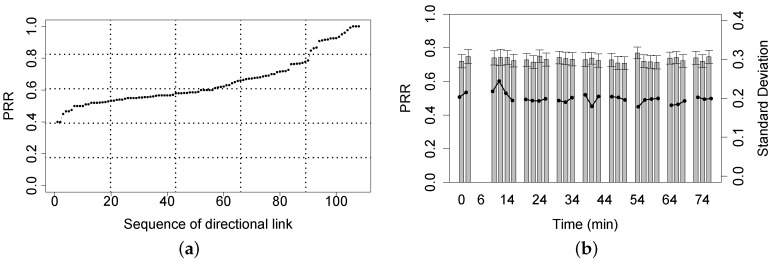
PRR performance of directional links. (**a**) Over each valid directional link; (**b**) Over all valid direction links against time.

Figure 15a shows the average PRR of each directional link in the Jiufeng experiment. We can see that most link PRRs range between 50% and 80%; good links whose PRR is greater than 90% account for only 14%; such an observation shows that in comparison with the single link experiment in the Bajia and the Campus forests, the network contains more probabilistic links in the mountain forest environment with irregularly-distributed, denser shrubs and trees, even though the transmit power is set to the highest level. Figure 15b plots the variation of the average link PRR as the time increases. Recall that the network updates its link quality (PRR) every two minutes; Figure 15b includes some missing PRR records due the the failed CTP paths, which cannot correctly deliver the link information to the sink. Clearly, during the experiment, the link quality stays dynamic, though being to a relatively small extent. The mean of PRR ranges from 70% to 80% and the standard variance of PRR from 16% to 25%.

**Figure 16:**
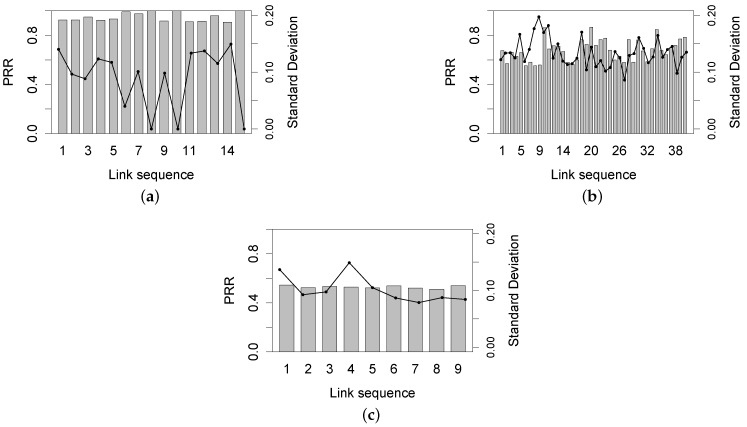
Symmetry variation of links with different link quality. (**a**) Good link; (**b**) Middle link; (**c**) Poor link.

Figure 16 examines the time-varying characteristic of link asymmetry. According to the average PRR in the whole experiment, we divide all of the two-way links into three categories: the poor link (PRR ≤55%), the middle link (55% < PRR < 90%) and the good link (PRR ≥90%). The standard variance of good links’ asymmetry is at most 15% and is often less than 10%, similar to the results of the Campus forest experiment; and this observation is also true for poor links. The asymmetry of middle links varies more frequently, leading to a relatively higher standard variance of at least 10%. These results demonstrate the possible relation between the link quality and the link asymmetry, yet with low confidence.

### 4.3. Exploring Link Correlation

**Figure 17:**
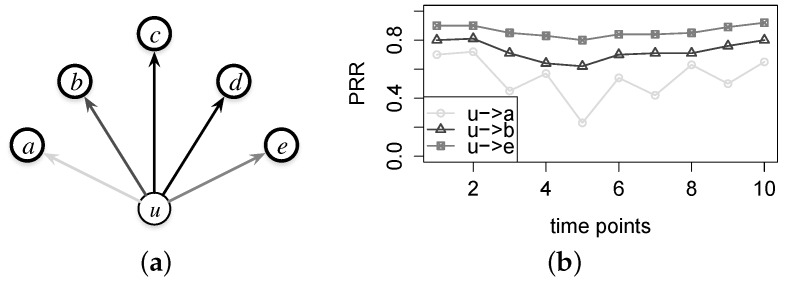
Conceptual illustration of the link correlation. (**a**) Demo of incident links; (**b**) Patterns of the PRR variations.

In this section, we propose a new performance metric for in-network links, the link correlation, which is potentially helpful to estimate or forecast the link performance and then can serve as a metric in network protocol designs. In general, the link correlation weighs the homogeneity of two links with a common sender in terms of the temporal variation of link quality. The left part of Figure 17 shows that sender *u* has five neighbors and then five corresponding directional links, $\ell_{u,a}$, $\ell_{u,b}$, …, and $\ell_{u,e}$; the right part shows the PRR variation as the time increases. It is clear that in Figure 17, the two PRRs of links $\ell_{u,b}$ and $\ell_{u,e}$ vary with a similar pattern, while neither of them is congenial with the PRR of link $\ell_{u,a}$. In such a case, the two links, $\ell_{u,b}$ and $\ell_{u,e}$, are regarded to be correlated; we can statistically estimate the PRR of $\ell_{u,e}$ by the PRR of $\ell_{u,b}$ if the PRR variation pattern of $\ell_{u,b}$ can be known a priori. We measure the correlation between two links by calculating the Pearson correlation coefficient of the PRRs of both links and define the correlation by Equation (3), where $\ell_{1}$ and $\ell_{2}$ are two directional links with a common sender, and $Prr_{1}$ and $Prr_{2}$ are two variables representing the PRR measurements for the two links, respectively.

(3) $$Corr\left(\ell_{1},\ell_{2}\right)=\left\{\begin{array}{c} 0,\;\;\;\mathrm{if}\;\sigma_{Prr_{1}}\cdot \sigma_{Prr_{2}}=0;\;\;\;\mathrm{otherwise}\\ \frac{E\left[Prr_{1}\cdot Prr_{2}\right]-E\left[Prr_{1}\right]\cdot E\left[Prr_{2}\right]}{\sigma_{Prr_{1}}\cdot \sigma_{Prr_{2}}} \end{array}\right.$$

Noticeably, the link correlation we emphasize here is different from the packet correlation considered in prior works [24,25,26]. The packet correlation is to profile the temporal correlation between two successive packets transmitted through a single link, and it is often calculated by a conditional probability. Specifically, the packet correlation could be employed in designing more efficient MAC schemes, e.g., adaptively deciding the back-off duration of retransmissions [24]. The link correlation, defined on two links with a common sender, is to quantify to what extent two such incident outgoing links perform similarly, i.e., to measure the possibility of deducing the performance of a link with the known performance of another sender-shared link. The link correlation metric can facilitate the decision making in the resource-constrained networking controls for wireless sensor networks. In Figure 17, for instance, if there exists significant correlation between the links $\ell_{u,a}$ and $\ell_{u,b}$ and the current link quality of $\ell_{u,a}$ is known, then we do not have to probe the link $\ell_{u,b}$ within at least a short window, say a few or tens of seconds; we can estimate the PRR of $\ell_{u,b}$ according to the PRR variation of $\ell_{u,a}$, thereby saving the precious system resources.

**Figure 18:**
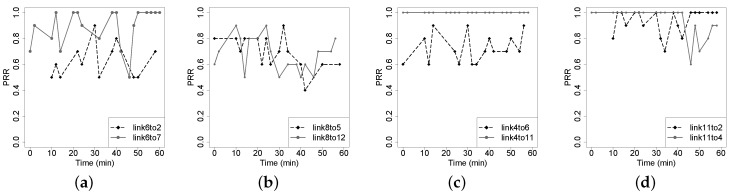
Demo of the link correlation measured in the Jiufeng experiment. (**a**) Correlation = 0.63; (**b**) Correlation = 0.28; (**c**) Correlation = 0; (**d**) Correlation = −0.4.

**Figure 19:**
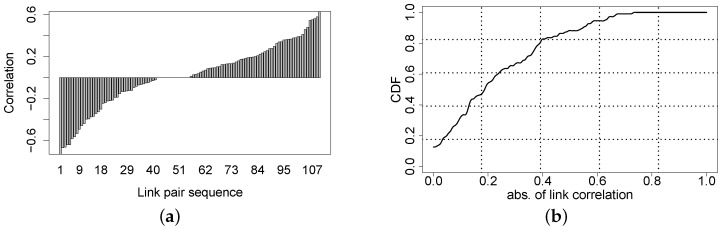
Correlation of all incident link pairs. (**a**) Correlation distribution; (**b**) CDF of the absolute value of correlation.

**Figure 20:**
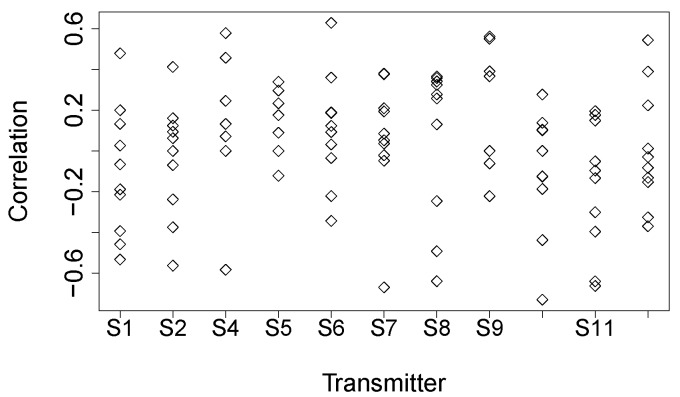
Correlation coefficient of any two links from each sending node.

Figure 18 shows four typically different link correlations. Figure 18a shows an obvious *good* correlation between links S6-S2 and S6-S7, while Figure 18d shows a *bad* correlation between links S11-S2 and S11-S4; there is no significant correlation between links S8-S5 and S8-S12 in Figure 18b, and there is no correlation between links S4-S6 and S4-S11 in Figure 18c. Figure 19a shows the correlation distribution of all of the pairs of incident links, and Figure 19b plots the cumulative distribution of the absolute value of link correlation. We find that among all of the pairs of links having a common sender, 91% of them are less than 0.6, and 80% of them are less than 0.4, in terms of the absolute correlation coefficient. For each sensor node S_*i*_, Figure 20 plots the distribution of the $Corr(\ell_{m},\ell_{n})$ where links $\ell_{m}$ and $\ell_{n}$ both start from sender S_*i*_. From Figure 20, we cannot see clear clusters of correlation coefficients for each sending node, and then, we cannot derive the convincing conclusion that the link correlation property is widespread in the low-power wireless networks deployed in forest environments. To some extent, this observation contradicts the packet correlation results achieved by several prior works that deploy sensor nodes either in a building or an open area. Even though not being a common behavior for low-power wireless networks deployed in forest, significant correlation between two incident links still exists and can often hold within a relatively long period, say at least a few minutes (see Figure 18a). In the complex forest environment, upper-level networking protocols could potentially depend on the link correlation information to achieve the resource-efficient estimation of the link quality or the link variation pattern.

## 5. Summary of Observations

By the comprehensive real-world experiments and comparative analyses, we summarize our observations and remarks as follows.

***Observation* 1**. The link performances are different, even under slightly different forest environments: the distributions of trees and understory shrubs, as well as vegetation will lead to different effects. Interestingly, a handful of shrubs (in the Campus forest) possibly constructively affect the link quality to some extent, in comparison with the forest with a “clean” understory (e.g., the Bajia forest of this paper). The wireless sensor deployment, therefore, does not have to circumvent the short and sparse shrubs if the line-of-sight links are hard to achieve in practice.

***Observation* 2**. In forests, the link quality usually degrades as the communication distance increases, but this degradation is nonlinear, indicating that sometimes, longer links may have better link quality than shorter ones. It is therefore unreasonable to estimate or compare the link qualities only by the link distance, as theoretical studies often do. According to the results of Figure 4, in particular, our experiments do not show obvious transitional regions (also called the grey region), which however, are found under open areas in [23,24]. Additionally, higher transmit power levels do not always imply higher link quality, and then, it may be undesirable for topology controls to improve the wireless network connectivity just by simply increasing the transmit power.

***Observation* 3**. For the time-constrained application [27], the efficient prediction of the link quality pattern is very important. The work [28] acclaims that RSSI is a good link estimator for low-power links. By comparing the variation patterns of the PRR, the RSSI and the LQI, however, we find that in forests, the LQI has a higher temporal correlation with the PRR, compared to the RSSI. Therefore, the LQI may be a better link estimator that can be employed by the upper layer protocols (such as MAC and routing) for in-forest deployment. In fact, the weak signal strength will not possibly lead to the failed packet reception; the variation of RSSI measurements mainly caused by the irregular obstacles in forests, therefore, cannot well reflect the real link performance, sometimes.

***Observation* 4**. In the experiment, the link in two directions is rendered significantly asymmetric in terms of the PRR, the RSSI and the LQI. In our experiments, the link symmetry does not obviously correlate with the link distance, which agrees with the previous empirical evaluations in clean environments. For a given communication distance, higher transmit powers of the two-way link usually lead to good link symmetry due to the good packet receptions. Different from prior results [28,29,30,31], however, no good link symmetry can be always observed either for the good link or for the poor link; the reason behind this may be that no significant transitional regions exist in the forest environment. We also find out the temporal variation of link asymmetry and different varying patterns for links with different PRRs. The link asymmetry is persistent and difficult to accurately predict. In total, for good or poor links, the asymmetry can be kept stable with light fluctuations, while for middle links, the asymmetry varies significantly within the relatively large range. Thus, careful considerations are needed in evaluating the low-power link reliability of the one-hop communication in the forest scenario; in practice, the data sent out over a link need an acknowledgment from the receiver for the purposes of confirmation, so the product of two-way PRRs of a link is often used to weigh the actual link quality.

***Observation* 5**. The temporal correlation between low-power wireless links with the common sender is less common, but exists. The correlation coefficient is not too significant: 91% of pairs of incident links are less than 0.6 in the correlation coefficient. Additionally, not all of the observed correlations are *good* (positive), and sometimes, they are *bad* (negative), unlike what is expected. The weak and irregular link correlation also indicates the obvious spatial difference among links. With such an observation, the upper layer protocols cannot reliably estimate the qualities of all the out-going links only according to the quality of a particular single link, unless they can assure significant correlation between two given incident links.

In summary, low-power wireless links deployed in forest environments are easily affected by the complex forest environment and then are both unreliable and extremely hard to precisely predict. Our experimental study discloses the importance of the following issues that need to be considered or addressed in practical wireless sensor networks deployed in forests: (1) how to model and evaluate the link validity with as effective and resource-efficient metrics as possible; (2) how to guarantee the reliable data delivery over the data-ack link of high asymmetry; (3) how to deploy wireless sensor nodes in forests such that the network topology can be kept efficiently connected with a high probability; (4) how to employ and schedule mobile sinks, such as mobile phones [9,32], to collect the sensory data, if the sensor network is unavoidably segmented due to poor links; and (5) how to achieve optimized cooperative designs across the link, the MAC and the routing layers [33] to further save the limited network energy.

## 6. Related Work

The link performance is always a major concern in wireless network applications. In particular, in wireless sensor networks, the low-power link is very easily affected by surrounding environments and rendered difficulty with respect to prediction. Investigation about how the low-power radio links behave has attracted more attention in the literature. In general, the characteristics of the radio link concerned by researches mainly include the link reliability, the link symmetry, the link regularity, the link performance estimator designs and their relationship with the environments, as well as the patterns of on-site deployment.

The salient work [34] investigates the link performance of wireless IEEE 802.11 networks, especially analyzing the link loss rate and providing some insights into the MAC and routing designs; the authors find that the wireless network as a whole is unreliable, and the link quality is not strongly correlated with the link distance and the signal-noise ratio. The key contribution in [34] is to emphasize the significance of carefully considering the link performance in wireless network applications. Cerpa et al. [23] empirically find that the realistic wireless link performance does not agree with the widely-used simulation models, and they statistically study the distribution of lossy radio links and evaluate their models through testbeds, which are deployed in indoor and outdoor environments. In [28], the authors initially evaluate the link RSSI that serves as a metric to profile link quality in wireless sensor networks. Through a precisely-controlled experiment (the environment is not explicitly given), the authors achieve different results: sometimes, the RSSI is a more desirable link metric than the pure LQI. Zhou et al. [29] model the irregularity presented by low-power radios and analyze the effect on the MAC and routing protocols. Zuniga et al. [15] theoretically study the transitional region (grey zone) issue in wireless sensor networks; and in their later work [30], they deeply explore the unreliability and the asymmetry of 915-MHz low-plow wireless links that are deployed in a building aisle and a football field, respectively, and then propose several simple numeric link-layer models.

Srinivasan et al. [24] deploy two wireless sensor networks both in the ceilings of the indoor scenario and show their experimental observations that support and dispute the ordinary assumptions on wireless communications. Particularly, the authors discuss the significance of real low-power link performance for the communication protocol designs. Their research suggests that there exists a clear gap between algorithm studies and real deployment. Baccour et al. [17] overview the literature and compare the observations and analyses of low-power links in prior works. The authors conclude that different wireless platforms and experimental environments will lead to different or even disputed outcomes, indicating the deficiency of some laboratory results and the necessity of investigating low-power links under certain real-world scenarios.

As mentioned in [17], the above results are often achieved in the case with the open sky view or the indoor environments with less obstacles and simple reflection surfaces. However, different environmental conditions might lead to different link behaviors even for the same wireless platform. In the recent few years, therefore, researchers have begun to investigate the low-power link of wireless sensor networks in several practical scenarios. Tang et al. [31] analyze the characteristics of IEEE 802.15.4 links in an indoor factory environment; they find that the path loss rate with factory-like surroundings is more significant and hard to model, in comparison with the counterpart under the open field. Mottola et al. [35] use a wireless sensor network to monitor road tunnels; they find that the LQI is more suitable than the RSSI in estimating the link performance, the link asymmetry is permanent at most times, and temporary link disruptions will occur when vehicular traffic exists. Liu [36] investigate the throughput variation of a wireless sensor network deployed in forest and present the temporal dynamics of low-power links. The works [37,38] studied the effect of temperature on the performance of low-power radio links. Marfievici et al. [39] evaluate the aggregated performance of all of the links and study the effect of outdoor environments on the physical layer of the link; especially, the authors analyze the effect of seasonal variations on the wireless links. Furthermore, the author in [40] collects the link measurements in target fields and then presents models for estimating the run-time link quality. These two papers offer a guide for our future work investigating the low-power link performance in forest environment. To monitor a Canadian boreal forest ecosystem, Rankine et al. [41] operate a wireless sensor network; they report that the forest environment is so complex that it is very hard to accurately model the link performance; they mainly focus on investigating the variation of link RSSI, and they believe that the canopy structure, the wind speed and the off leaves possibly impact the link performance.

There have been some efforts at investigating the physical layer characteristics of in-forest wireless links [42,43,44,45]. Raman et al. [42] measure the physical layer of wireless mesh networks and indicate that the interference, rather than the multipath fading effect, is the major cause of dynamic links. In [44], a propagation model for the ZigBee link with the transmit power of 18 dBm is fit according to the experiment data obtained in a forest with relatively sparse trees. The work [46] studies the wireless sensor network link over a vegetarian area and then proposes two path loss models with empirical parameters. Azpilicueta et al. [47] conduct a wireless sensor network experiment under the inhomogeneous vegetation scenario and they qualitatively confirm the effect of vegetation topology on the link performance. The works in [48,49] model the path loss of VHF band wireless links penetrating forests. The error properties of IEEE 802.15.4 links in industrial environments are studied by Barac et al. [50]; the authors show that for the low-power links, the link errors caused by multipath fading and attenuation are different, in terms of pattern, from that caused IEEE 802.11 interference. Those physical-layer works attempt to deduce the radio propagation parameters in practical scenarios; the results might be helpful for simulation experiments, but cannot be easily employed by the upper layer protocols at runtime, especially for the capability-limited wireless sensor networks.

## 7. Conclusions

This paper has presented and analyzed the evaluation results of wireless low-power links (compliant with the IEEE 802.15.4 standard) in two in-city forests and one on-mountain forest. The evaluation uses the PRR, the RSSI and the LQI as the basic metrics for link quality and investigates the in-forest low-power link characteristics from the following aspects: the effects of link distance and transmit power level on the link quality, the time-varying characteristics of link quality and link asymmetry, the empirical radio propagation model and the correlation between incident links. This paper also summarizes the observations and gives some open research issues in deploying wireless sensor network in forest environments. As suggested in [51,52], the weather conditions or seasonal factors possibly affect the performance of the low-power wireless link, which are not involved in this paper. Our future work is to deploy and operate a large-scale low-power link wireless sensor network in a forest for a longer term and to conduct more comprehensive investigations.
